# Why you should share your data during a pandemic

**DOI:** 10.1136/bmjgh-2021-004940

**Published:** 2021-03-15

**Authors:** Emily R Smith, Valerie J Flaherman

**Affiliations:** 1 Department of Global Health, The George Washington University, Washington, DC, USA; 2 Department of Pediatrics, University of California San Francisco, San Francisco, California, USA

**Keywords:** systematic review, maternal health, epidemiology, COVID-19, cohort study

**Summary box**

- Prospective meta-analyses and other innovative approaches for pooling data from multiple studies have the potential to expedite the pace at which knowledge is produced, especially for observational or surveillance data.
- In working to pool published and unpublished data over the past year, we have encountered resistance to data sharing from scientists accustomed to a more traditional approach.
- Common concerns and misunderstandings are that participating in prospective data pooling: (1) might be considered to be (unethical) publication of overlapping data; (2) may render study-specific manuscripts less novel, less prestigious or less appealing to high-impact journals; and (3) it may be unethical to share or analyse data repeatedly while data collection is ongoing.
- We review the likely source of these concerns and argue there are not any robust reasons to avoid sharing data for appropriately designed, collaborative projects that can advance global health.

The devastating impact of the COVID-19 pandemic can be partly attributed to a lack of evidence to inform effective prevention and treatment. The global scientific community has been racing against time to rapidly generate such evidence. Prospective meta-analysis (PMA) and other innovative approaches for pooling data from multiple studies are increasingly used and have the potential to expedite the pace at which knowledge is produced, especially for observational or surveillance data. However, investigators engaged in these innovative projects must leave behind some traditional practices of academic research.^1^ For that reason, the use of PMA or other real-time pooling efforts may meet resistance from individual investigators dubious about the professional, ethical and utilitarian implications of such innovation.

We have previously proposed that a sequential PMA offers a useful approach to rapidly generate policy and practice-relevant guidance; our group is currently engaged with investigators working in 21 countries to pool data related to SARS-CoV-2 infection during pregnancy.^2^ While the PMA process requires commitment from investigators and some effort to harmonise data collection elements, it also provides substantial potential benefits related to rapid dissemination of information. Through collaboration, serially updated PMAs allow results to be shared well before adequate sample sizes are available for individual studies and can therefore rapidly inform public health policy decisions such as those needed for the management of the pandemic.

In working to pool published and unpublished data, we have encountered resistance to data sharing from scientists accustomed to a more traditional approach. Although many investigators, especially those working in low-income and middle-income countries or with prior international collaboration experience, have readily agreed to participate, some investigators working in academic settings in high-income countries have expressed doubts. We address some of the most common misunderstandings related to observational data pooling that we have encountered.

## One common misunderstanding is the concern that participating in prospective data pooling efforts will be perceived as publishing overlapping data

This concern is unfounded for two reasons. First, while the importance of avoiding duplicative publication of data from a single participant in two or more reports can hardly be overstated^3^, there is a straightforward method for handling this type of data overlap, which is to disclose it in publications. Individual patient data meta-analyses, whether prospective or retrospective, inherently achieve this disclosure by clearly identifying the sources of data and reanalysing it for purposes of pooling. Second, the International Committee of Medical Journal Editors (ICMJE) guidance on overlapping publications (developed prior to the current pandemic but highly relevant in light of current circumstances) strongly encourages dissemination of data in the context of a public health emergency, without concern of detriment to future publication, and it urges editors to give priority for publication to any study that has shared crucial information.^4^ The ICMJE’s prescient endorsement of data sharing in any setting of urgent scientific need strongly supports participation in well-designed pooling activities during the current pandemic.

Another frequently expressed misunderstanding is that manuscripts analysing previously shared data may be perceived as less novel, less prestigious or less appealing to high-impact journals than those presenting data not previously shared. While this was a compelling concern in the past, the recent deluge of data published on preprint servers like medRxiv—and the clear success of those preprints in terms of Altmetric score and subsequent publication—demonstrate changing attitudes about prepublication results dissemination.^5^ In fact, high-impact journals, including those in the BMJ family of journals, strongly encourage such sharing.^6^ Contributing data to a pooled analysis, when transparently disclosed, has thus not shown itself to be prejudicial to publication in journals following such principles. Furthermore, publishing a single study, including all data points and a thorough discussion of the context, methods, strengths and limitations of the study, should still be valued for providing different insight than that of a pooled analysis. Investigators with concerns about a publication’s perceived prestige should be reassured that participating in meta-analyses is generally thought to increase the visibility and impact of individual studies.^7^

## Some investigators have argued that it is unethical to share or analyse data repeatedly while data collection is ongoing

While this may be true for randomised controlled trials—where underpowered interim analyses may bias future data collection, cause participant withdrawal or hamper future recruitment—ethical considerations regarding surveillance and other observational data are wholly different. The objectives of observational research are not related to study-supplied interventions; interim data analysis has little potential to introduce detrimental bias. Indeed, the most common way repeated data analysis might influence observational studies of COVID-19 would be by providing evidence to inform policies or guidelines that benefit future participants of such a study. This appears to be an argument in favour of ongoing analysis of observational data. Surveillance is a core component of public health science; ongoing collection, timely dissemination and linkages to public health practice are essential.^8^ If surveillance identifies modifiable factors that are successful in preventing or ameliorating disease, this is considered a major success and public health good.^9^

Effective response to the COVID-19 pandemic necessitates revisiting historical conventions. While established norms such as concealing data until publication may have benefits during times of stable knowledge generation, these norms have significant costs including preventing rapid dissemination of crucial knowledge. In the current public health environment—characterised by massive increases in global morbidity and mortality—contributing data to pooled analyses is a contribution to the global good. Answers to basic epidemiological questions regarding COVID-19 infection are urgently needed worldwide. We argue there are not any robust reasons to not share data given appropriately designed, collaborative projects that can advance global health.
